# Combination of knockout serum replacement and plasma rich in growth factors does not support in vitro spermatogenesis in mice

**DOI:** 10.1038/s41598-025-16502-7

**Published:** 2025-08-26

**Authors:** Seyyed Amir Moradian, Sajed Khaledi, Zahra Amirkhani, Mansoureh Movahedin

**Affiliations:** 1 Department of Reproductive Biology, Faculty of Advanced Medical Sciences, Tabriz University of Medical Sciences, Tabriz, Iran; 2 Department of Anatomical Sciences, Faculty of Medical Sciences, Tarbiat Modares University, Tehran, Iran; 3 Department of Nursing, School of Nursing, Larestan University of Medical Sciences, Larestan, Iran; 4 Student Research Committee, Larestan University of Medical Sciences, Larestan, Iran

**Keywords:** In vitro spermatogenesis, Testicular tissue culture, Culture medium, PRGF, KSR, Spermatogonial stem cell, Cell biology, Stem cells

## Abstract

Cancer treatments can lead to infertility, particularly in prepubertal boys who cannot preserve sperm before therapy. In vitro spermatogenesis offers a promising strategy for fertility preservation in this population by enabling the development of sperm from immature testicular tissue under controlled conditions. This study investigates the effects of a novel culture medium containing plasma rich in growth factors (PRGF) and knockout serum replacement (KSR) on in vitro spermatogenesis. Testicular tissues from five-day-old male NMRI mice were cultured in either a medium containing 5% KSR + 5% PRGF or a control medium containing 10% KSR for 42 days. Histological analysis revealed significant degeneration of peripheral seminiferous tubules in the 5% KSR + 5% PRGF group compared to the control. Gene expression analysis showed reduced levels of spermatogenesis markers (*Plzf*, *Tekt1*, *Tnp1*) and the proliferation marker *Ki67*, alongside elevated expression of the pro-apoptotic marker *Bax*. Immunofluorescence confirmed fewer spermatogonial stem cells (PLZF), spermatocytes (SYCP3), and proliferating cells (Ki67), with complete absence of post-meiotic marker ACRBP in the 5% KSR + 5% PRGF group. Additionally, higher Bax and lower Bcl-2 fluorescence intensities were observed in this group. These findings indicate that a medium supplemented with 5% KSR and 5% PRGF is ineffective for supporting complete in vitro spermatogenesis and may promote apoptosis. Importantly, these results provide insights into culture system design for future applications in fertility preservation strategies for prepubertal cancer patients at risk of gonadotoxicity.

## Introduction

Spermatogenesis is a complex and tightly regulated process in which spermatogonia develop into mature spermatozoa. It begins shortly before puberty under the influence of pituitary gonadotropins and continues throughout life^1^. Cancer treatments such as chemotherapy and radiotherapy can impair spermatogenesis, often resulting in infertility. This issue is especially critical in prepubertal boys, whose testes contain only immature germ cells and therefore lack the option of sperm cryopreservation before treatment^2^. Approximately 200,000 children under 15 years are diagnosed with cancer annually^3^, with improved survival rates reaching nearly 80% due to advancements in treatments^4^. However, infertility following treatment remains a major quality-of-life issue, affecting around 30% of survivors^5–7^.

Adult male patients can cryopreserve sperm prior to gonadotoxic treatments, but prepubertal patients lack this option because their testes contain only immature germ cells and Sertoli cells, not mature sperm^7^. Therefore, developing an effective culture system that can support in vitro spermatogenesis from immature testicular tissue is a priority for fertility preservation in this population^4,8^. Such systems could also benefit men with non-obstructive azoospermia (NOA)^9^.

In response to this clinical need, significant efforts have been made over the past decades, to develop optimized testicular tissue culture media. Early attempts using Eagle’s medium supplemented with amino acids and bovine serum were unsuccessful in supporting complete spermatogenesis, as germ cells failed to progress beyond the pachytene spermatocyte stage^10^. Subsequent studies identified key supplements such as pyruvate, non-essential amino acids, and hormones (e.g., FSH) that improved spermatogenic progression but still did not achieve full spermatogenesis^11^.

Building upon these findings, in 1993, Boitani et al. cultured testicular tissues from 9-day-old rats in EMEM supplemented with various vitamins and hormones, demonstrating that FSH was essential for the progression of type A spermatogonia to pachytene spermatocytes over three weeks. In contrast, other supplements such as LH, testosterone, and vitamins A, C, and E did not promote this progression^12^.

A major breakthrough came with a study by Sato et al. demonstrated that supplementation with 10% Knockout Serum Replacement (KSR) could support complete spermatogenesis in mouse testicular tissue cultures, achieving functional sperm production^13^. Despite these advances, subsequent research highlighted that even 10% KSR media were less efficient than in vivo spermatogenesis^14–16^ and showed strain-dependent variability in outcomes^17^, underscoring the need for further media optimization.

In recent years, attention has turned to incorporating growth factor-rich supplements such as platelet-rich plasma (PRP) and its derivative, plasma rich in growth factors (PRGF), into culture media. PRGF provides a concentrated source of bioactive molecules, including platelet-derived growth factor (PDGF), glial cell line-derived neurotrophic factor (GDNF),vascular endothelial growth factor (VEGF), and insulin-like growth factor-1 (IGF-1), which have been shown to enhance stem cell proliferation, migration, and differentiation in various tissues^18–23^.

Prior studies on PRP have indicated its positive impact on the viability and differentiation of spermatogonial stem cells, with some reports highlighting the upregulation of post-meiotic gene expression, while others emphasize pre-meiotic activation^24,25^. Notably, optimal outcomes in such studies were generally observed at low concentrations, such as 5% PRP, suggesting a concentration-dependent effect^24^.

In our previous work^26^, we systematically evaluated various concentrations of PRGF (5%, 10%, and 20%) for testicular tissue culture. Among them, 5% PRGF was found to best preserve seminiferous tubule integrity over a 14-day period, performing comparably to 10% KSR. Furthermore, 42-day cultures supplemented with 5% PRGF supported spermatogenesis up to the formation of flagellated sperm. This was accompanied by enhanced expression of key spermatogenesis markers and reduced apoptotic signaling, indicating its potential to support germ cell development in vitro. Nevertheless, further optimization is needed to increase the efficiency of producing a higher number of mature, flagellated sperm.

In this context, combining KSR with PRGF may offer a synergistic approach by providing both a defined serum replacement and a rich mixture of growth factors to optimize the in vitro environment. Although some studies have reported beneficial effects of such combinations in germ cell cultures of other species^27^, their specific impact on mouse testicular tissue remains unexplored. Despite growing interest in PRGF for various regenerative purposes, its application in testicular organ culture remains largely unexplored and lacks standardized protocols. This prompted the present study, which aims to systematically evaluate the combined effects of 5% PRGF and 5% KSR on in vitro spermatogenesis in mouse testicular tissue.

## Materials and methods

### Ethics declarations

All experimental protocols were reviewed and approved by the Institutional Animal Care and Use Committee (IACUC) of Tarbiat Modares University (Approval ID: IR.MODARES.REC.1399.043). All procedures involving animals were performed in accordance with relevant national guidelines and institutional regulations for animal welfare, and are reported in compliance with the ARRIVE guidelines.

For human sample collection, venous blood was obtained from healthy adult volunteers following written informed consent. The protocol was conducted in accordance with the ethical principles outlined in the Declaration of Helsinki, and was approved by the Ethics Committee of Tarbiat Modares University.

### Animals and housing conditions

Neonatal (5-day-old) male NMRI mice were obtained from the Royan Institute (Tehran, Iran). Animals were housed under a 12-h light/dark cycle at a controlled temperature of 22 ± 2 °C and 55 ± 5% humidity, with ad libitum access to water and standard chow. All procedures were conducted in compliance with national and institutional animal care regulations.

### Preparation of PRGF

A total of 300 mL of peripheral blood was collected from three healthy male adult volunteers using 50 mL tubes containing 3.8% sodium citrate. Blood samples were centrifuged at 2500 g for 4 min to separate plasma, followed by a second centrifugation at 5000 g for 5 min to isolate platelet-rich fractions. The upper buffy coat layer (Fraction F2), with the highest platelet concentration, was pooled and activated by adding 10% CaCl₂ (50 µL/mL). The mixture was incubated in a 40 °C water bath for 90 min to induce gel formation. The resulting supernatant was centrifuged at 8000 g for 10 min, aliquoted, and stored at − 80 °C. Before use, PRGF aliquots were thawed and re-centrifuged (10,000 g, 10 min, 4 °C) to remove residual clots^28^.

### Culture media composition

The base medium consisted of α-MEM (Gibco, Thermo Fisher Scientific) supplemented with 60 ng/mL progesterone (Invitrogen, UK), 30 ng/mL β-estradiol (Pepro Tech, USA), 20 ng/mL epithelial growth factor (EGF) (Pepro Tech, USA), 10 ng/mL human basic fibroblast growth factor (bFGF) (Pepro Tech, USA), 10 ng/mL human glial cell line-derived neurotropic factor (GDNF) (Pepro Tech, USA), 10 ng/mL leukemia inhibitory factor (LIF) (Royan, I.R.I), 100 IU/mL penicillin, 100 µg/mL streptomycin, and 50 µg/mL gentamicin. For the experimental group, 5% PRGF and 5% KSR (Invitrogen) were added. The control group received 10% KSR without PRGF.

### Testicular tissue culture

Testes from 5-day-old mice were dissected under sterile conditions and sectioned into fragments of 1–3 mm. Tissue fragments were placed atop 1.5% agarose gel blocks (10 × 10 × 5 mm) pre-equilibrated in culture medium. Cultures were maintained at 34 °C in a 5% CO_2_ incubator with medium changes every two days. The culture duration was 42 days, corresponding approximately to the full cycle of mouse spermatogenesis.

### Histological evaluation

After 42 days, tissues were fixed in Bouin’s fixative (3 h), embedded in paraffin, sectioned (5 µm), and stained with hematoxylin and eosin (H&E). Morphological analysis was conducted under light microscopy to evaluate tubule structure and degeneration.

### RNA extraction and real-time PCR

Total RNA was extracted using RNX-Plus™ (CinnaGen, Iran) from five pooled samples per group. RNA was quantified spectrophotometrically and treated with DNase I. First-strand cDNA synthesis was performed using the RevertAid™ kit (Thermo Fisher) with oligo(dT) primers. Gene expression of *Plzf*, *Tekt1*, *Tnp1*, *Ki67*, *Bax*, and *Bcl-2* was assessed using SYBR Green-based qPCR (Applied Biosystems) (Table 1). β-Actin was used as the housekeeping gene. Relative expression was calculated using the 2^−ΔΔCT^ method. The expression level of each target gene in the control group (testicular tissue cultured with 10% KSR) was set to 1, and fold changes in the experimental group were calculated accordingly. Statistical significance was considered at *p* < 0.05.

**Table 1:** Primer sequences used for gene expression analysis of spermatogenesis stages and related cellular functions.

Target	Cell type / function	Accession number	Sequence (5′–3′)
*Plzf*	Spermatogonia	NM_001033324.3	F: GCTGCTGTCTCTGTGATGG R: GGGCTGATGGAACATAGGGG
*Tekt1*	Spermatocyte	NM_011569	F: CACCAGGAAGTCTCAGAGCGAT R: GTAAGCAGGTCATCCGTCTGGT
*Tnp1*	Spermatid	NM_009407.2	F: TGTGATGCGGCAATGAGC R: CGACTGGGATTTACCCACTC
*Ki67*	Proliferation marker	NM_001081117	F: GAGGAGAAACGCCAACCAAGAG R: TTTGTCCTCGGTGGCGTTATCC
*Bax*	Apoptosis (pro)	NM_017059.2	F: TTTGCTACAGGGTTTCATCCAG R: GTCCAGTTCATCGCCAATTC
*Bcl2*	Anti-apoptotic	NM_016993.1	F: GAGAGCGTCAACAGGGAGAT R: ACAGCCAGGAGAAATCAAACA
*β-actin*	Housekeeping gene	NM_007393	F: TCCCTGGAGAAGAGCTACG R: GTAGTTTCGTGGATGCCACA

### Immunofluorescence staining

Testicular tissues cultured for 42 days were fixed overnight at 4 °C in 4% paraformaldehyde prepared in phosphate-buffered saline (PBS). Paraffin-embedded sections (5 μm) were deparaffinized, rehydrated, and subjected to antigen retrieval by heating in 10 mM trisodium citrate buffer (pH 6.0) using a microwave at 600 W for 20 min. Sections were then washed with TBS containing 0.03% Triton X-100 (Sigma-Aldrich, Germany), and non-specific binding was blocked by incubation in 1% BSA (in TBS) for 2 h at room temperature.

Subsequently, sections were incubated overnight at 4 °C with primary antibodies against PLZF (spermatogonial stem cell marker), SYCP3 (spermatocyte marker), ACRBP (post-meiotic spermatid marker), Ki67 (proliferation marker), Bax (pro-apoptotic), and Bcl-2 (anti-apoptotic), all at 1:100 dilution (PLZF, SYCP3, ACRBP, Bax, and Bcl-2 from Santa Cruz Biotechnology, USA; Ki67 from Elabscience, China). After washing, sections were incubated with Cy3-conjugated goat anti-mouse IgG secondary antibody (1:100, Elabscience) for 1 h in the dark.

Nuclei were counterstained with 4’,6-diamidino-2-phenylindole (DAPI; 1:200, Sigma-Aldrich) for 5 min. Sections were dehydrated through graded ethanol, cleared in xylene, mounted with coverslips, and examined under a fluorescence microscope (Olympus BX50, Germany).

For quantification, four random fields per sample were captured at 400 × magnification. The number of PLZF-, SYCP3-, ACRBP-, and Ki67-positive cells per seminiferous tubule was counted, while the mean fluorescence intensity of Bax and Bcl-2 was measured using ImageJ software (NIH, Bethesda, MD, USA), following the method described by Leite et al^29^.

### Statistical analysis

Data were analyzed using GraphPad Prism 4 (GraphPad Software, USA). Comparisons between groups were made using unpaired two-tailed t-tests. Prior to analysis, normality of data distribution was confirmed using the Shapiro-Wilk test, and equality of variances was assessed using Levene’s test. Results are reported as mean ± standard deviation (SD), and *p*-values < 0.05 were considered statistically significant.

## Results

### Histological evaluation

**Fig. 1:**
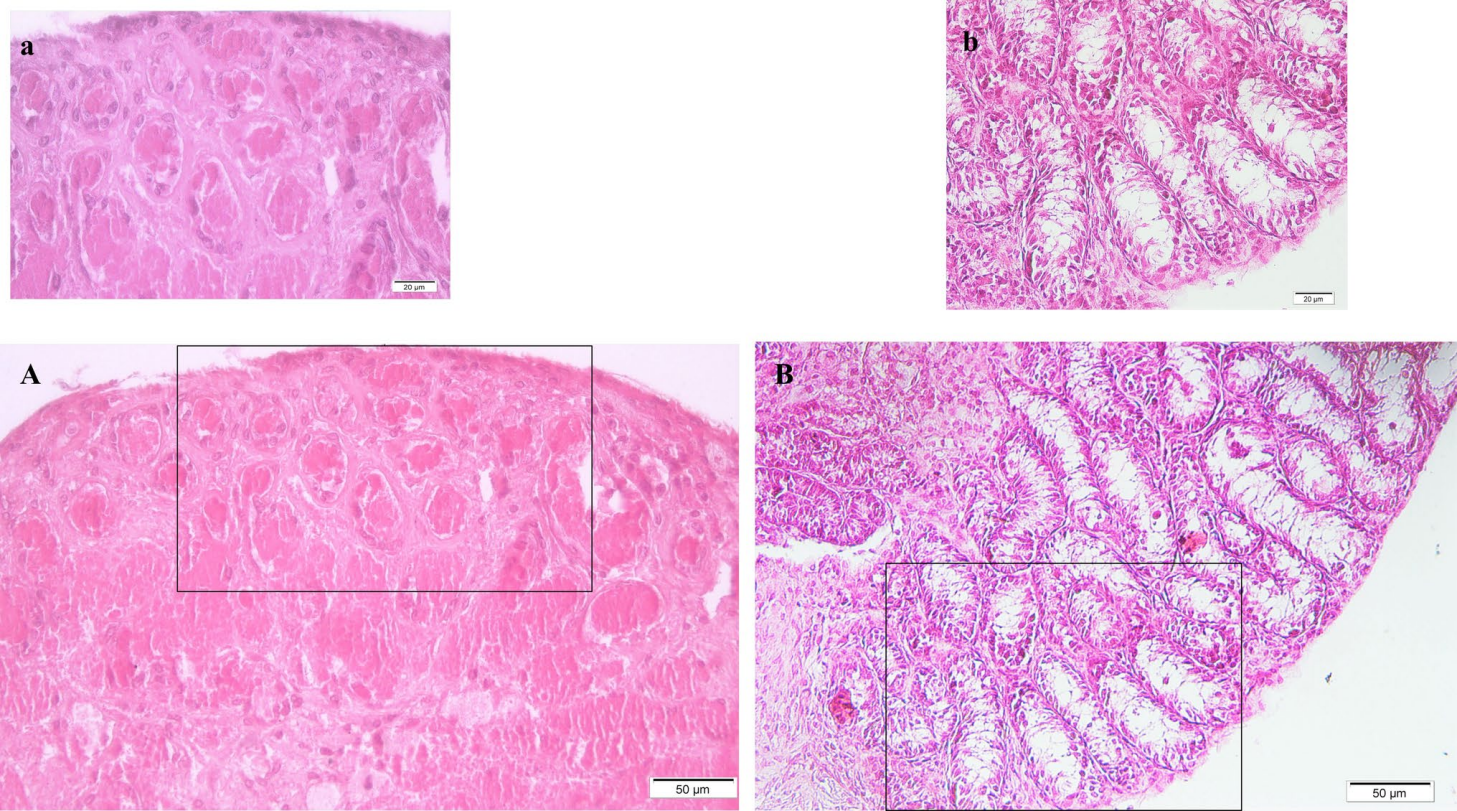
Representative histological sections of testicular tissues cultured for 42 days in two different medium conditions. (**A**) Cross-sectional overview of tissue cultured with 5% KSR + 5% PRGF, showing extensive central necrosis and structural degeneration of seminiferous tubules. (**a**) Higher magnification of the peripheral region shown in (**A**), revealing loss of germ cell layers, detachment from basement membrane, and increased pyknotic nuclei. (**B**) Cross-sectional overview of tissue cultured with 10% KSR, demonstrating relatively preserved seminiferous tubule architecture. (**b**) Higher magnification of the peripheral region shown in (**B**), demonstrating relatively preserved epithelial organization and partial maintenance of germ cell layering, despite mild disorganization in some tubules. All sections were stained with hematoxylin and eosin (H&E); scale bars = 50 μm (**A** and **B**), 20 μm (**a** and **b**).

After 42 days of culture, histological analysis revealed that testicular tissues maintained in 5% KSR + 5% PRGF exhibited extensive central necrosis and pronounced degeneration of seminiferous tubules (Fig. 1A). The peripheral tubules also showed marked structural disorganization, with reduced germ cell cohesion, loss of epithelial layering, diminished attachment to the basement membrane, and an increased number of pyknotic and apoptotic nuclei. As a result, the outer regions of the tissue were largely depleted of viable cells (Fig. 1a).

In contrast, tissues cultured in 10% KSR exhibited relatively preserved morphology (Fig. 1B). A torus-like peripheral zone of seminiferous tubules was maintained, with partially retained germ cell stratification and intact basement membranes, despite the presence of central necrosis and structural disorganization in the core region (Fig. 1b).

### Gene expression analysis of spermatogenesis, proliferation, and apoptosis markers

**Fig. 2:**
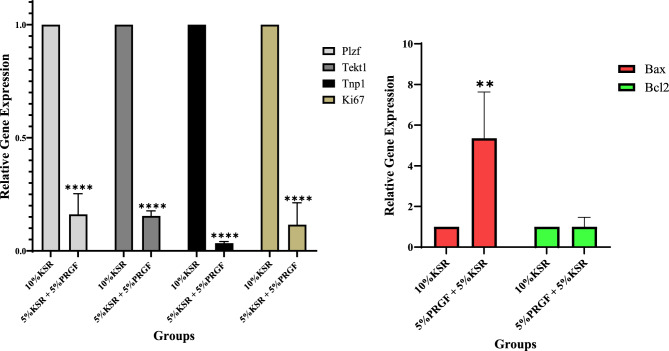
Relative mRNA expression levels of spermatogenesis- and apoptosis-related genes in testicular tissues cultured for 42 days in either 10% KSR or 5% KSR + 5% PRGF media. Genes analyzed included Plzf (spermatogonia marker), Tekt1 (spermatocyte marker), Tnp1 (spermatid marker), Ki67 (proliferation marker), Bax (pro-apoptotic), and Bcl-2 (anti-apoptotic). Gene expression was measured by real-time PCR, and data were normalized to the housekeeping gene β-actin. Expression in the 10% KSR group was set as the calibrator (fold change = 1). Statistical comparisons were performed using unpaired two-tailed t-tests. Significance is indicated as: *P* < 0.01, **P < 0.0001 for 10% KSR vs. 5% KSR + 5% PRGF.

Quantitative real-time PCR analysis revealed significant alterations in the expression of genes associated with spermatogenesis, proliferation, and apoptosis between the two groups (Fig. 2). In the 5% KSR + 5% PRGF group, the expression levels of *Plzf* (spermatogonia), *Tekt1* (spermatocytes), *Tnp1* (post-meiotic spermatids), and *Ki67* (proliferation marker) were all significantly downregulated compared to the 10% KSR group (*P* < 0.0001 for all; Fig. 2). This pattern indicates a broad suppression of germ cell development and mitotic activity under KSR + PRGF treatment.

Conversely, expression of the pro-apoptotic gene *Bax* was significantly increased in the KSR + PRGF group (*P* < 0.01), while *Bcl-2*, an anti-apoptotic gene, showed no significant difference between the two conditions. The elevated *Bax* expression, in the absence of a compensatory rise in *Bcl-2*, suggests a shift in the apoptotic balance toward cell death in the KSR + PRGF-treated tissues.

### Quantitative immunofluorescence analysis

**Fig. 3:**
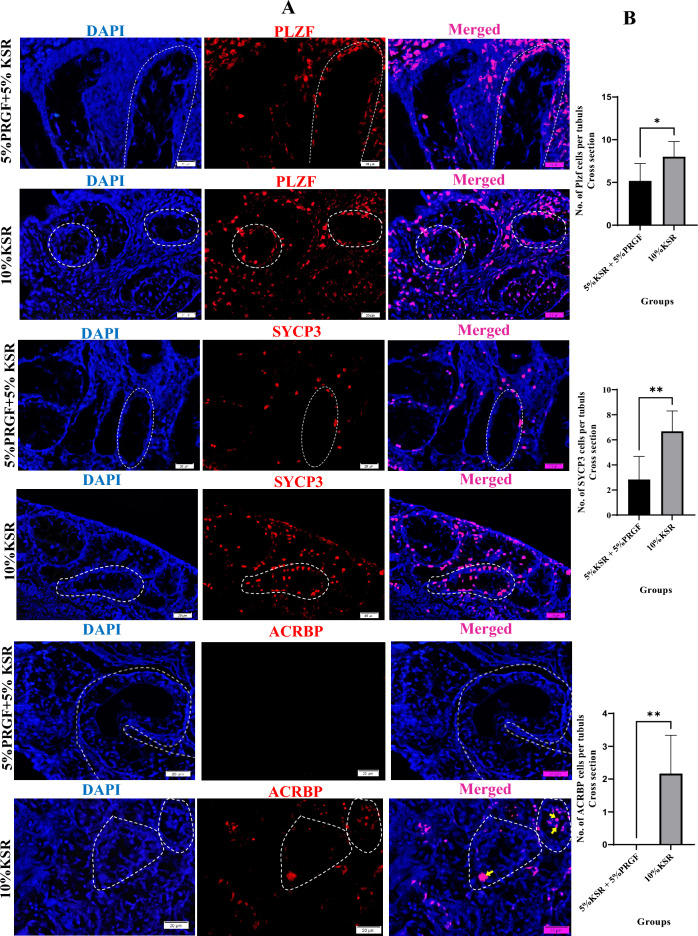
Immunofluorescence staining and quantitative analysis of germ cell markers in testicular tissues cultured for 42 days in media containing either 5% KSR + 5% PRGF or 10% KSR. (A) Representative immunofluorescence images showing PLZF-positive (PLZF⁺) spermatogonial stem cells, SYCP3⁺ spermatocytes, and ACRBP⁺ post-meiotic spermatids (red). Cell nuclei were counterstained with DAPI (blue). Yellow arrowheads indicate ACRBP⁺ spermatids. Dashed white lines indicate the basal lamina of seminiferous tubules. Scale bar = 20 μm. (B) Quantification of the average number of PLZF⁺, SYCP3⁺, and ACRBP⁺ cells per tubule. Tissues cultured in 10% KSR showed significantly higher counts compared to the 5% KSR + 5% PRGF group. Data are presented as mean ± standard deviation (SD). Significance is indicated by *P ≤ 0.05, **P ≤ 0.01.

**Fig. 4:**
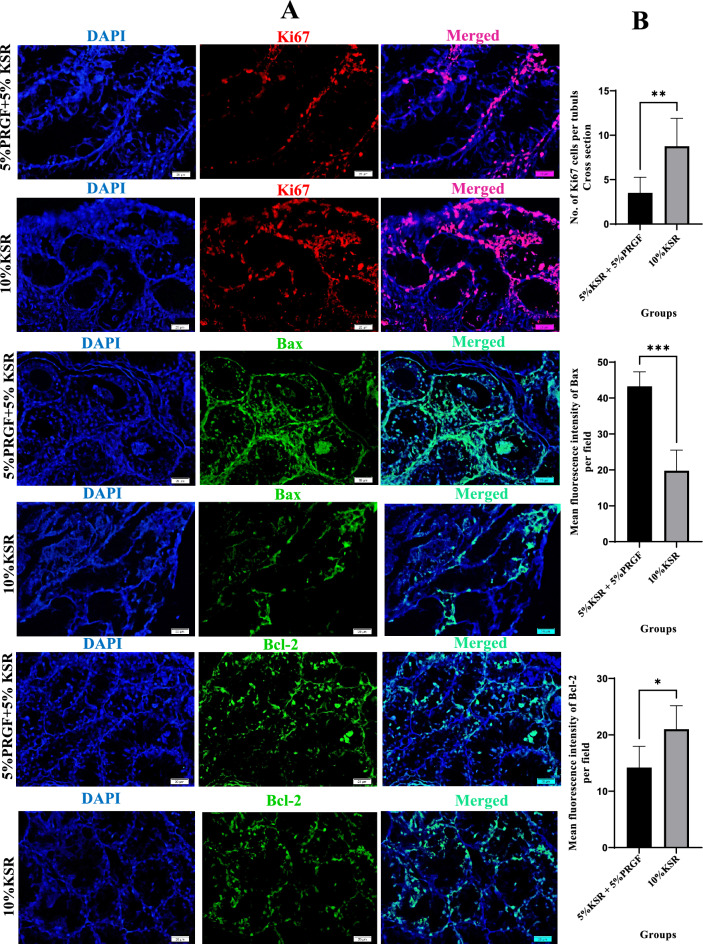
Immunofluorescence staining and quantitative analysis of Ki67, Bax, and Bcl-2 protein expression in testicular tissues cultured for 42 days in media containing either 5% KSR + 5% PRGF or 10% KSR. (**A**) Representative images showing immunofluorescence staining for the proliferation marker Ki67 (red), the pro-apoptotic marker Bax (green), and the anti-apoptotic marker Bcl-2 (green). Cell nuclei were counterstained with DAPI (blue). Scale bar = 20 μm. (**B**) Quantitative analysis revealed significantly higher Ki67 expression in the 10% KSR group, indicating enhanced proliferative activity. Conversely, tissues cultured with 5% KSR + 5% PRGF exhibited a significant increase in Bax expression and a decrease in Bcl-2 expression, suggesting enhanced apoptotic activity. Data are presented as mean ± standard deviation (SD). Significance levels: *P ≤ 0.05, **P ≤ 0.01, ***P ≤ 0.001.

Immunofluorescence staining further supported the molecular findings, as shown in Figs. 3A and 4A. Tissues cultured in 5% PRGF + 5% KSR exhibited significantly fewer PLZF-positive spermatogonial stem cells per seminiferous tubule (*p* = 0.0285), as well as a notable reduction in SYCP3-positive spermatocytes (*p* = 0.0034) compared to the 10% KSR group (Fig. 3B). These data confirm early-stage germ cell depletion at both premeiotic and meiotic levels.

Importantly, ACRBP-positive post-meiotic spermatids were entirely absent in the KSR + PRGF group, indicating that germ cell development was arrested prior to completion of meiosis. In contrast, 10% KSR-supported cultures showed robust ACRBP expression within multiple tubules (*p* = 0.0011), demonstrating successful progression to the spermatid stage under this condition (Fig. 3B).

Proliferative activity, as measured by Ki67-positive cell counts, was also significantly diminished in the KSR + PRGF group (*p* = 0.0011), consistent with the observed downregulation of *Ki67* transcript levels (Fig. 4B).

Fluorescence intensity analysis of apoptotic markers revealed that tissues in the KSR + PRGF group exhibited significantly elevated Bax signal intensity (*p* = 0.0005), alongside reduced Bcl-2 expression (*p* = 0.027), confirming increased apoptotic stress at the protein level in the KSR + PRGF-treated cultures (Fig. 4B).

## Discussion

In this study, we assessed whether a combination of 5% PRGF and 5% KSR could support in vitro spermatogenesis in neonatal mouse testicular tissue. Our results demonstrate that this formulation was inadequate for preserving seminiferous tubule architecture and sustaining germ cell differentiation throughout the extended culture duration.

Histological examination revealed extensive degeneration, characterized by central necrosis, peripheral disorganization of seminiferous tubules, and loss of cell adhesion. These architectural disruptions were paralleled by molecular alterations, including reduced expression of spermatogenic markers (*Plzf*, *Tekt1*, *Tnp1*) and the proliferation marker *Ki67*, alongside elevated *Bax* expression. Immunofluorescence analysis further confirmed depletion of PLZF + spermatogonia, SYCP3 + spermatocytes, and a complete absence of ACRBP + post-meiotic cells. Increased Bax and decreased Bcl-2 and Ki67 labeling indicate that this formulation may trigger an apoptotic shift through molecular imbalance, compromising germ cell viability and progression.

The observed detrimental effects may arise from the interaction between biologically distinct components: PRGF, a human-derived plasma concentrate rich in growth factors^30^, and KSR, an animal-derived chemically defined supplement optimized for embryonic stem cell maintenance^31^. While each component supports cell survival under specific conditions, their combined presence may disrupt the finely balanced testicular microenvironment.

An excessive or imbalanced supply of bioactive compounds from both PRGF and KSR—including lipids, vitamins, hormones, and particularly growth factors—may overwhelm endogenous signaling pathways^32^. Examples include PDGF, VEGF, IGF-1, HGF, bFGF, and TGF-β1. These factors, though beneficial at physiological concentrations, can elicit paradoxical responses when overabundant, such as oxidative stress, aberrant intracellular signaling, and apoptosis^33–38^. Notably, TGF-β and HGF can activate stress-associated pathways (e.g., p38 MAPK) and upregulate cell cycle inhibitors (e.g., p21Waf1, p27Kip1), leading to cell cycle arrest and apoptosis^39–42^. Previous studies have also reported that supraphysiological levels of LIF or bFGF impair spermatogonial proliferation^43^. These molecular pathways may underlie the increased Bax expression and decreased Bcl-2 levels observed in our study, and help explain the degeneration of testicular architecture in the KSR + PRGF-treated group.

In our previous study^26^, we demonstrated that 5% PRGF alone was effective in supporting spermatogenic progression and maintaining seminiferous structure. However, the addition of 5% KSR to this concentration negated its beneficial effects. This observation aligns with previous reports indicating that excessive or unbalanced supplementation with serum components such as PRP can exert detrimental effects on cell viability. The dose-dependent cytotoxicity of PRP has been documented in various systems. For example, PRP at 40–60% reduces proliferation in adipose-derived mesenchymal stem cells, with 20% being optimal^38^. Similarly, high concentrations (≥ 50%) suppress periodontal cell viability^44^, and complete PRP (100%) induces cytotoxicity in osteoblasts and ligament cells^45^. These findings highlight the necessity of carefully titrating bioactive supplements in culture systems.

Parallel evidence exists for KSR. A study by Liu et al. demonstrated that 5% KSR provided insufficient support for testicular cultures, while 15% induced atrophy after initial growth. At 15% KSR, rapid initial growth slowed after two weeks, with shrinkage observed by the fourth week—likely due to cellular toxicity. In contrast, 10% KSR yielded optimal outcomes, underscoring the importance of concentration balance and highlighting the dose-dependent risk of cellular toxicity associated with excessive KSR supplementation^46^.

Collectively, our findings underscore the sensitivity of in vitro spermatogenesis to the precise composition and dosage of culture medium components. A mechanistic understanding of how specific growth factor interactions affect spermatogonial stem cell fate is essential for developing optimized protocols that enable complete spermatogenic progression. This knowledge will be critical for advancing in vitro fertility preservation strategies.

Despite providing critical insights, this study has certain limitations. The absence of mechanistic pathway validation and intermediate time-point assessments limits our understanding of the dynamic cellular responses to PRGF and KSR. Future studies incorporating transcriptomic or proteomic profiling, alongside time-course analyses, are warranted to unravel these complex interactions.

## Conclusion

This study demonstrated that culturing testicular tissues with a combination of 5% KSR and 5% PRGF for 42 days resulted in significant structural and functional deterioration compared to the standard 10% KSR condition. The observed degeneration was marked by central necrosis, disrupted tubule organization, downregulation of spermatogenesis- and proliferation-related genes, and increased pro-apoptotic signaling (Bax). Quantitative immunofluorescence further confirmed reduced numbers of spermatogonial stem cells, spermatocytes, and proliferating cells, alongside increased Bax and decreased Bcl-2 expression. These findings underscore the need for careful optimization of growth factor supplementation in culture systems aiming to support complete spermatogenesis in vitro. This is particularly critical for developing personalized fertility preservation strategies in prepubertal patients who are vulnerable to gonadotoxic therapies.

## Data Availability

The data supporting the findings of this study are available from the corresponding authors upon reasonable request.
